# Characterization of the complete chloroplast genome of *Bupleurum hamiltonii* N. P. Balakr. (Apiaceae) and its phylogenetic implications

**DOI:** 10.1080/23802359.2020.1870902

**Published:** 2021-02-09

**Authors:** Gaixia Zhang, Hui Wang, Xin Yin, Weijun Kong, Qiuling Wang, Yi Chen, Jianhe Wei, Jinxin Liu, Xinwei Guo

**Affiliations:** a Institute of Medicinal Plant Development, Chinese Academy of Medical Sciences and Peking Union Medical College, Beijing, China; b Institute of Sericulture, Chengde Medical University, Chengde, China; c Chengde Academy of Agricultural and Forestry Sciences, Chengde, China; d College of Continuing Education, Guizhou University of Traditional Chinese Medicine, Guiyang, China; e Hebei Key Laboratory of Study and Exploitation of Chinese Medicine, Chengde Medical University, Chengde, China

**Keywords:** *Bupleurum hamiltonii*, chloroplast genome, phylogenetic, identification

## Abstract

*Bupleurum hamiltonii* has a thin, wood, grayish-yellow root and is reputed to possess medicinal value. This study employs the *de novo* method using high-throughput sequencing data to assemble the complete chloroplast genome for this species. The total *B. hamiltonii* genome is 155,320 bp in length, containing a large single-copy region of 85,280 bp, a small single-copy region of 17,468 bp, a pair of inverted repeat regions of 26,286 bp, and has a GC content of 37.8%. The genome encodes 113 unique genes, of which there are 79 protein-coding, 30 tRNA, and four rRNA genes. In addition, 18 genes contained introns, *petB*, *petD*, and *rpl16* are coded for by two exons, and *rps12* is identified as a trans-splicing gene. Phylogenetic analysis results strongly suggest that *B. hamiltonii* is closely related to *B. marginatum*. This study provides valuable genetic information to facilitate reliable identification.

*Bupleurum* L. is a large genus in the family, Apiaceae, with about 180 species widely distributed in the North Temperate Zone (She and Watson 2005). In China, 44 species, 17 varieties, and seven forma of this genus have been reported, of which 25 species, eight varieties, and three forma have been recorded as resources for the traditional Chinese herbal medicine Radix Bupleuri (‘Chai hu’) (Pan et al. 2002). However, the official original plants listed for ‘Chai hu’ in Chinese Pharmacopeia only include *B. chinense* DC and *B. scorzonerifolium* Willd. Given the high species diversity and reputable medicinal value of this genus, the taxonomy assignment, chemical profiling, and biological activity of many species in the genus, *Bupleurum* have received considerable attention (Wang et al. 2011b). Several easily confused species are used as folk medicine in some local areas (Liu 2008; Yang et al. 2007). As an annual or short-lived perennial plant with a thin root and characteristic fruit (She and Watson 2005), *Bupleurum hamiltonii* N. P. Balakrishnan is commonly documented as ‘Xiao chai hu’ (Liu 2008; Ma et al. 2010; Wang et al. 2011a), which constitutes a similar name as the famous composite formula ‘xiao chai hu tang.’ In addition, *B. chinense,* widely cultivated in Gansu, is also commonly called ‘Xiao chai hu’ (Zhang 2020), which may lead to the misuse of *B. hamiltonii* and *B. chinense*.

Fresh *B. hamiltonii* leaves were collected from Supu Town, Qianxi County, Bijie City, Guizhou Province (N26°59′39.75″, E106°21′29.02″). The voucher specimen with the number of HPCH0003 was placed in the herbarium at the Institute of Medicinal Plant Development, Chinese Academy of Medical Sciences and Peking Union Medical College (IMD). Total genomic DNA was isolated from the leaves using modified CTAB-based extraction (Porebski et al. 1997). The quantity and quality of the DNA were examined using the Qubit 4.0 (Thermo Fisher Scientific Inc., USA). After the total DNA was sheared, the TruSeq DNA PCR-free library preparation guide was used to generate a library with an average insert size of 270 bp. The Illumina NovaSeq platform was employed to conduct high-throughput sequencing, and approximately 3.4 GB of raw data were generated with 150 bp paired-end read lengths. Low-quality reads and the sequencing adapter were filtered using Trimmonmatic v0.38 (Bolger et al. 2014), and only the ‘paired’ output files where both reads survived processing were used for the subsequent analysis. The *de novo* assembly of the complete chloroplast genome was completed using the NOVOPlasty toolkit (Dierckxsens et al. 2016), and the average genome sequencing depth was 516. The genome annotation for protein-coding, rRNA, and tRNA genes was performed with CPGAVAS2 (Shi et al. 2019) on the website www.herbalgenomics.org/cpgavas2. The gene and repeat element map of the annotated *B. hamiltonii* chloroplast genome was generated synchronously during the annotation process.

The chloroplast genome sequence of *B. hamiltonii* is 155,320 bp in length and shows a typical quadripartite structure like most angiosperm plants, with two reverse repeated regions (IRa and IRb) of 26,286 bp in length. The repeat regions divide the entire chromosome into two single-copy regions, namely a small single-copy region (SSC) and a large single-copy region (LSC) with 17,468 bp and 85,280 bp, respectively (GenBank accession no. MW262986). The total GC content of the chloroplast genome was 37.8% and encodes 113 unique genes. Of these, 79 were protein-coding, 30 represented tRNA, and four are rRNA genes. Furthermore, 18 genes contain introns, 15 (nine protein-coding and six tRNA genes) contain one intron and three (*rps12*, *ycf3*, and *clpP*) two introns. Moreover, *petB*, *petD*, and *rpl16* are coded by two exons, while *rps12* is identified as a trans-splicing gene.

To confirm the phylogenetic location of *B. hamiltonii* in the genus *Bupleurum* of the Apiaceae, a total of 37 complete chloroplast genomes were used for the phylogenetic analysis using the Maximum Likelihood (ML) method with RAxML v8.0.0 (Stamatakis 2014) and 1000 bootstrap replicates. *Panax ginseng* of the family, Araliaceae, and *Cornus capitata* of the family, Cornaceae, were designated as the outgroups. Based on the results of the phylogenetic analysis, the nine species of the genus *Bupleurum* were fully supported in a monophyletic clade with a bootstrap value of 100 (Figure 1), which was consistent with previous studies (Yang et al. 2007). The ML tree showed that *B. hamiltonii* and *B. marginatum* were resolved in the same clade, while *B. chinense*, *B. scorzonerifolium,* and five additional species of the genus *Bupleurum* were sister in position. The complete chloroplast genome of *B. hamiltonii* will provide valuable genetic information for further study involving genetic diversity, especially regarding reliable identification.

**Figure 1.**
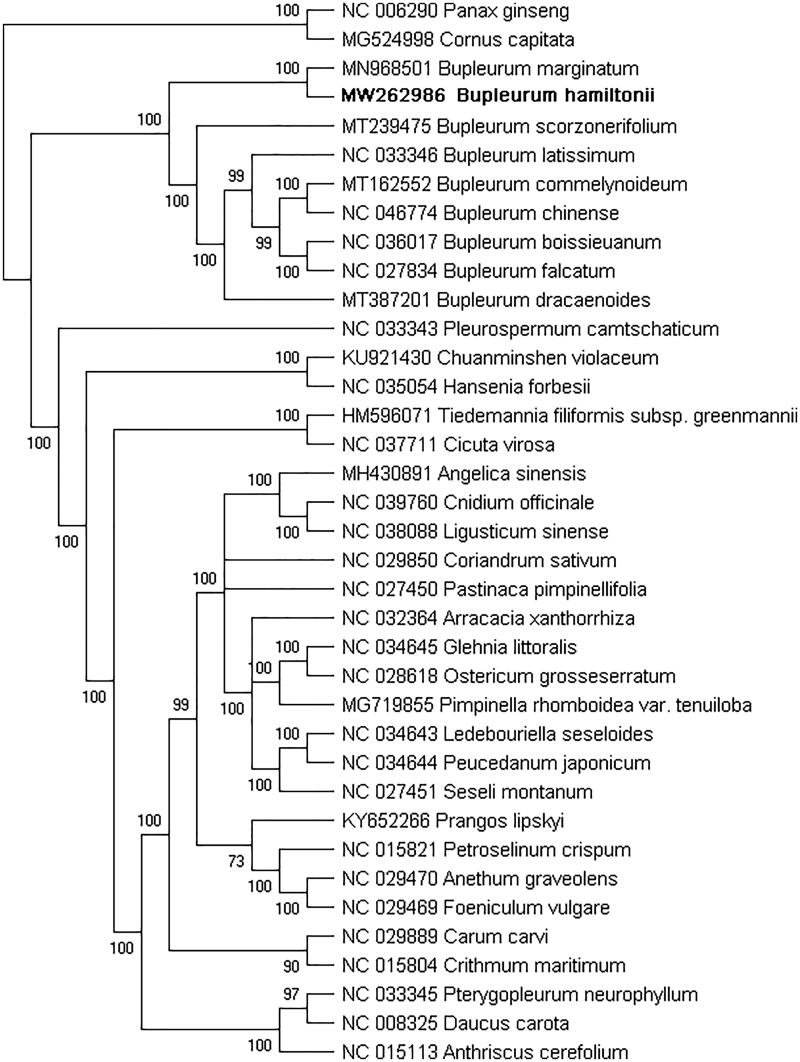
Phylogenetic relationships of *Bupleurum hamiltonii* and 36 complete chloroplast genomes. Bootstrap support values are given on the branches.

## Data Availability

Chloroplast data supporting this study are openly available in GenBank at nucleotide database, https://www.ncbi.nlm.nih.gov/nuccore/ MW262986, Associated BioProject, https://www.ncbi.nlm.nih.gov/bioproject/ PRJNA682316, BioSample accession number at https://www.ncbi.nlm.nih.gov/biosample/ SAMN16987561 and Sequence Read Archive at https://www.ncbi.nlm.nih.gov/sra/ SRR13189612.
